# Real-World Transition to a Preservative-Free Fixed Combination of Dorzolamide/Timolol: Impact on the Ocular Surface Microenvironment, Safety, Tolerability, and Efficacy

**DOI:** 10.3390/medicina62010184

**Published:** 2026-01-16

**Authors:** Ana Sanseau, Arturo Burchakchi, Fernando Cataldi, Héctor Fontana, Alejo Peyret, Giselle Rodríguez, Ailín Fantacone, María Silvia Passerini, Javier F. Casiraghi

**Affiliations:** 1 Hospital J. M. Ramos Mejía, Ciudad Autónoma de Buenos Aires C1221ADC, Argentina; 2 Hospital Italiano de Buenos Aires, Ciudad Autónoma de Buenos Aires C1199ABB, Argentina; 3 Clínica de Ojos Dr. Nano, Buenos Aires B1636CSS, Argentina; 4 Hospital Santa Lucía, Ciudad Autónoma de Buenos Aires C1232AAC, Argentina; 5 Hospital Durand, Ciudad Autónoma de Buenos Aires C1405DCS, Argentina; 6 Laboratorios POEN S.A.U., Ciudad Autónoma de Buenos Aires C1407BDR, Argentina; 7 Hospital de Clínicas, Ciudad Autónoma de Buenos Aires C1120AAR, Argentina

**Keywords:** glaucoma, dorzolamide, timolol, preservative-free, ocular surface disease, ocular surface microenvironment

## Abstract

*Background and Objectives*: This study evaluates the safety, tolerability, and efficacy of preservative-free Dorzolamide 2%-Timolol 0.5% (PF-DT), with a focus on improving the ocular microenvironment in a real-world transition setting. *Materials and Methods*: A prospective, multicenter, open-label study involving thirty patients with dry eye disease previously treated with BAK-DT was conducted. Participants were transitioned to PF-DT, and evaluated at weeks 4, 12, and 24. The primary endpoint was the Ocular Surface Disease Index (OSDI) score. Secondary outcomes included Break-Up Time (BUT), Schirmer test results, corneal staining, conjunctival hyperemia, intraocular pressure (IOP), and patient satisfaction. *Results*: Twenty-five patients completed the study. The OSDI improved from 21.5 to 12.5 (*p* < 0.001), with 60.0% of patients showing improvement and 52.0% achieving complete symptom resolution. Among eyes with corneal staining, 78.4% demonstrated a reduction of at least one grade, and 50.0% of those with conjunctival redness showed similar improvement. By week 24, 78.0% exhibited no corneal staining, and 50.0% had no conjunctival redness. BUT increased from 5.0 to 7.0 (*p* < 0.01), while IOP decreased by 1 mmHg (*p* < 0.01). Satisfaction regarding comfort (≥80%) and handling (≥50%) was high, with 88.0% preferring PF-DT. *Conclusions*: Transitioning to PF-DT improved ocular surface health while maintaining IOP control, supporting the benefits of preservative-free formulations in restoring microenvironment homeostasis and enhancing tolerability and patient satisfaction.

## 1. Introduction

The ocular surface is a finely balanced and complex system that relies on the integrity of its structural and functional components [1]. All tissues, specialized cells, extracellular matrix elements, small molecules, and the resident microbiome interact to form the ocular surface microenvironment [1]. Lifestyle factors increasingly influence ocular health by altering this homeostatic balance, potentially leading to the development of ocular surface disease (OSD) [1,2,3].

OSD encompasses a variety of conditions affecting the ocular surface, with dry eye disease (DED) being the most frequent [2]. Topical medication can contribute to the development of OSD through allergic, toxic, and immuno-inflammatory mechanisms [4]. Since glaucoma treatment often requires the chronic use of multiple topical medications, OSD is highly prevalent in this patient population, with reported rates ranging from 42% to 59% [5,6,7,8,9,10].

OSD significantly impacts the quality of life of glaucoma patients, causing symptoms such as foreign body sensation (33.5%), itching (22.8%), hyperemia (33.3%), photophobia (24.0%), blurred vision (17.3%), and pain (10.4%) [8]. Consequently, this affects treatment adherence, leading to higher intraocular pressure (IOP) levels, IOP fluctuation [11,12,13] and, consequently, compromise the therapeutic success of glaucoma management, with the risk of the underlying chronic disease progressing toward irreversible optic nerve damage [8,14]. Furthermore, topical glaucoma medication is also associated with an increased risk of failure in subconjunctival glaucoma surgery [15].

Although it has been demonstrated that OSD may result from the toxic effects of the active ingredients in the formulation, in most cases it is primarily caused by the cumulative toxic effect of their preservatives. Benzalkonium chloride (BAK), the most widely used preservative [11], is a quaternary ammonium that produces damage to corneal and conjunctival epithelial cells, loss of conjunctival goblet cells, tear film instability and epithelial barrier dysfunction [16,17]. BAK also reduces corneal nerve density and delays corneal wound healing [17,18]. In addition, BAK induces lymphocyte infiltration and increases the levels of inflammatory markers in ocular tissues [19,20]. It is important to note that the most frequently used concentration is at least four times the threshold concentration at which toxicity occurs [16].

Byun et al. have demonstrated that these effects can be partially reversed upon removal of BAK exposure [17]. Furthermore, TFOS DEWS II strongly recommends the use of preservative-free (PF) products when available, to limit the harmful side effects related to their use [17,21]. In addition to reducing toxicity on the ocular surface, minimizing the preservative load in ophthalmic formulations promises to improve treatment adherence, quality of life, and doctor-patient relationship [21,22,23].

BAK containing Dorzolamide 2%-Timolol 0.5% fixed combination (BAK-DT) was the first FDA-approved fixed combination for IOP reduction and it remains one of the most widely used medications worldwide [11]. Recently, a PF fixed combination of Dorzolamide 2%-Timolol 0.5% (PF-DT) was developed, which required replacing the traditional packaging with a specially designed container to maintain product sterility without the need for preservatives. The aim of this study was to evaluate the safety, tolerability, and efficacy of a new PF Dorzolamide 2%-Timolol 0.5% ophthalmic solution, compared with a BAK-containing regimen, with a focus on improving the ocular surface microenvironment in a real-world transition setting.

## 2. Materials and Methods

A phase IV multicenter, open-label, single-arm, prospective clinical trial was conducted in Argentina between March 2023 and October 2024. The study was carried out in the private offices of the participating ophthalmology professionals. This study was designed and conducted according to the criteria set by the Declaration of Helsinki and applicable local regulations (Resolution 1480/11 of the Ministry of Health of Argentina). The study protocol was approved by the research ethics committee of the Swiss Argentine Clinic and Maternity before the start of the study. Written informed consent was obtained from all patients prior to participation in this study.

Subjects had to meet the following inclusion criteria to be eligible for participation in the clinical trial: (1) male or female ≥18 years of age; (2) who provided written informed consent; (3) with primary open-angle glaucoma or bilateral ocular hypertension; (4) who had been under treatment with BAK-preserved Dorzolamide 2%-Timolol 0.5% solution for at least the previous 6 months; (5) corneal thickness measured by pachymetry within the range of 520 to 580 µm; (6) ocular surface disease index (OSDI) score greater than 13 points; (7) intraocular pressure < 20 mmHg in both eyes and (8) at least one of the following clinical signs of dry eye disease: tear Break-Up Time (BUT) < 10 s, Schirmer test < 5 mm/5 min, or positive corneal staining with more than 5 points of corneal erosion.

Participants were excluded from the study if they met any of the following criteria: (1) history of respiratory diseases; (2) history of cardiac diseases; (3) history of severe renal insufficiency (CrCl < 30 mL/min); (4) any ocular pathology that according to the investigator’s judgment could compromise the safe administration of the study medication or the safe participation in the study; (5) history of progressive retinal or optic nerve disease other than glaucoma; (6) active ocular inflammatory or infectious conditions; (7) progressive cataracts; (8) comorbidities of the ocular surface other than OSD; (9) eyelid disorders; (10) oral use of carbonic anhydrase inhibitors or beta-blockers; (11) oral use of medications that interact with Dorzolamide or Timolol; (12) use of any other topical ocular medication, in addition to the anti-glaucoma medication, especially those for dry eye treatment such as lubricants, corticosteroids, and immunomodulators; (13) regular use of contact lenses; (14) active autoimmune diseases; (15) history of laser keratorefractive procedures, corneal surgery, or corneal surface surgery, within 6 months prior to the baseline visit; (16) history of any intraocular or extraocular laser surgery or surgery on either eye within the 6 months prior to the baseline visit; (17) severe loss of central visual field in either eye (defined as a sensitivity of ≤10 dB in at least two of the four closest test points to the fixation point); (18) known hypersensitivity to any component of the study medications; (19) pregnant or lactating women.

At the baseline visit (day 0), patients were switched from BAK-preserved Dorzolamide 2%-Timolol 0.5% (BAK-DT) solution to the new PF Dorzolamide 2%-Timolol 0.5% (Glaucotensil^®^ TD LC, POEN S.A.U, Buenos Aires, Argentina) (PF-DT). The dosing schedule with the new formulation was the same as patients had been using with BAK-preserved Dorzolamide 2%-Timolol 0.5% solution (one drop twice a day). The new formulation is presented in an ophthalmic squeeze dispenser, designed to maintain sterility without preservatives (Aptar Pharma, Crystal Lake, IL, USA).

Patients were evaluated at baseline (day 0), visit 1 (4 weeks), visit 2 (12 weeks), and visit 3 (24 weeks). The primary efficacy endpoint was the Ocular Surface Disease Index (OSDI) score change after 24 weeks of treatment. The OSDI was selected as the primary endpoint because it reflects patient-perceived symptoms, which was a key outcome of interest in this real-world study evaluating ocular surface changes after preservative withdrawal.

The obtained OSDI score was categorized according to the severity of dry eye disease as follows: normal = 0–12; mild = 13–22; moderate = 23–32; and severe = 33–100 [24,25]. Secondary outcomes assessed at all visits included IOP measurement, conjunctival hyperemia, corneal staining, BUT, tear volume and best-corrected visual acuity. IOP was measured in millimeters of mercury (mmHg) using a calibrated Goldmann Applanation Tonometer (GAT) after having applied topical anesthesia and fluorescein staining. Conjunctival hyperemia was assessed using the CCLRU (Cornea and Contact Lens Research Unit) scale [26]. It was graded from 0 to 4, with 0 representing the total absence of hyperemia, 1 indicating a very mild degree, 2 mild, 3 moderate, and 4 severe hyperemia. Corneal staining was evaluated using fluorescein staining and slit lamp examination. The punctate epithelial erosions (PEEs) stained with fluorescein were counted. A score from 0 to 3 was assigned based on the PEE count, with 0 representing PEE absence, 1 indicating 1–5 PEEs, 2 indicating 6–30 PEEs, and 3 indicating more than 30 PEEs. An additional point was added if the PEE occurred in the central portion of the cornea, one or more filaments were observed anywhere on the cornea or the corneal staining pattern included linear structures anywhere on the cornea. The maximum possible total score was 6. The number of patients in each category and the median at each visit were assessed. Satisfaction regarding comfort was subjectively evaluated at all visits using the following question: “Are you satisfied with the comfort of the ophthalmic product you are using?”. Satisfaction regarding the handling of the ophthalmic squeeze dispenser was also subjectively evaluated at all visits using the following question: “Are you satisfied with the handling of the new LC dropper?”. Finally, patient preference was assessed at visit 3 with the following question: “Would you continue with the treatment you are currently using, or do you prefer the previous one?”. No standardized definition of comfort or satisfaction was given to participants, as the aim was to capture their spontaneous perceptions and subjective experience with the treatment.

Sample size was calculated based on the primary endpoint at 24 weeks. The initial calculation determined that a sample size of 22 patients would provide a statistical power of 90% or greater at a 0.05 significance level to detect a difference of more than 12 points in the OSDI score (common SD, 15). This value was deliberately chosen because a 12-point change in OSDI reflects a clinically meaningful improvement in ocular surface symptoms from the patient’s perspective. Accordingly, the sample size was designed to ensure adequate power to detect changes that are not only statistically significant but also clinically relevant. Considering a 15% dropout rate, the required sample size was adjusted to 25 patients.

Analyses were conducted at the eye level, including both eyes of each patient when eligible, rather than restricting the analysis to the worse eye. This approach was chosen to maximize the use of available data and to better reflect routine clinical practice, where both eyes are typically managed simultaneously. Analyses followed the intention-to-treat principle, including all patients with baseline and at least one follow-up assessment, using available data without imputation for missing values. No formal sensitivity analyses were performed.

The normality of the data was evaluated using the Kolmogorov–Smirnov test or the Shapiro–Wilk test. Demographic data were analyzed using the *t*-test and the chi-square test. Median and 95% confidence interval (95% CI) were presented for continuous variables, along with percentages for categorical variables. The Friedman test for paired samples was used to compare medians and the distribution of ordinal variables across visits. Bonferroni correction was applied to adjust the *p*-values. McNemar test was used to compare paired proportions of signs and symptoms absence across visits. Analyses were performed considering *p* < 0.05 as a statistically significant value. As per the intention-to-treat (ITT) principle, all patients with outcome measurements at baseline and at least one of the follow-up visits were included in the analyses. All analyses were conducted using IBM SPSS statistical software (version 22.0, IBM Corp.).

## 3. Results

**Figure 1:**
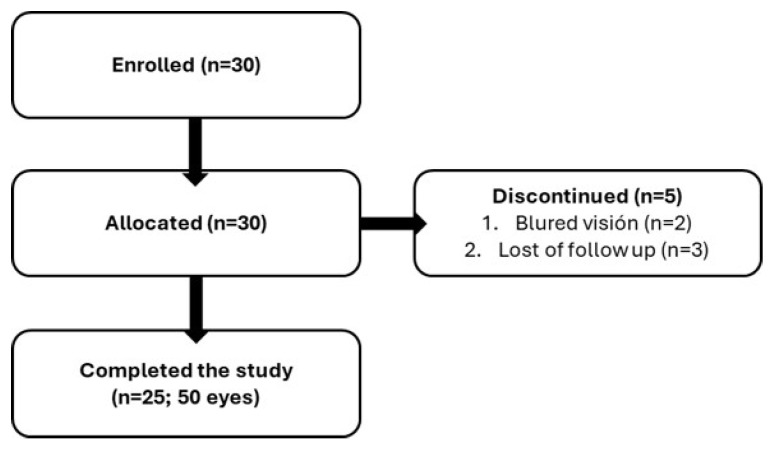
Flowchart of patient disposition throughout the clinical trial (n = number of patients).

A total of 30 patients from 6 ophthalmology centers were enrolled, and 25 patients (50 eyes) completed the study according to the protocol, of whom 19 were female (63.3%) and 11 were male (36.7%) (*p* = 0.273), with a mean age of 68.8 ± 16.68 years (Table 1, Figure 1). Two patients (6.7%) discontinued treatment because of blurred vision. Three patients (10.0%) were lost to follow-up. Best-corrected visual acuity did not differ from baseline to weeks 4, 12, and 24 (*p* = 0.793).

**Figure 2:**
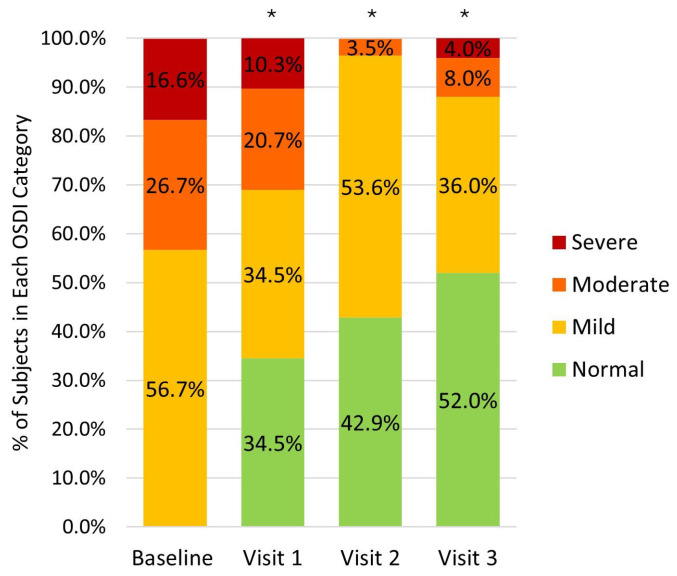
Percentage of subjects categorized by symptoms severity at baseline and follow-up visits. * Indicates *p* < 0.001 for patients with no symptoms between baseline and follow-up visits.

A statistically significant decrease in the OSDI score was observed at all follow-up visits (Table 2). At visit 3, 88.0% of patients experienced a reduction in the OSDI score, and 60.0% showed at least a one-level decrease. In addition, 52.0% of patients reported no OSD-related symptoms at that visit (Figure 2).

At baseline, 96.7% of patients presented with altered BUT (<10 s), 76.7% with altered Schirmer test values (<10 mm/5 min), 71.6% with positive corneal staining and 85.0% with conjunctival hyperemia. At week 24, 62.5% of eyes with initially altered BUT showed an increase of at least 1 s. BUT significantly improved at visits 2 and 3 (Table 2). No significant differences were observed in Schirmer test values between baseline and follow-up visits (Table 2). However, 67.5% of eyes that initially had altered Schirmer test values showed an increase of at least 1 mm/5 min at week 24.

**Figure 3:**
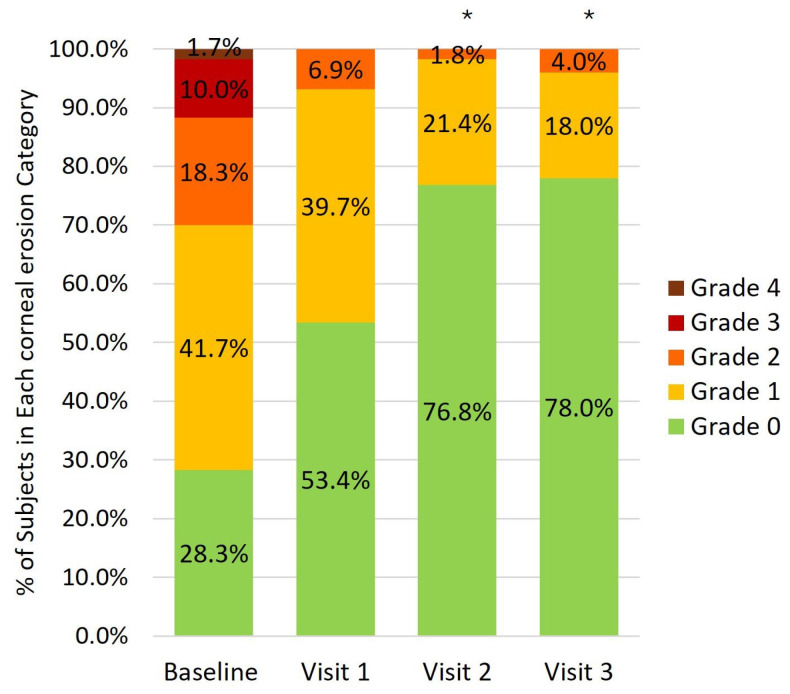
Percentage of eyes in each punctate corneal erosion category at baseline and follow-up visits. * Indicates *p* < 0.01 for patients with grade 0 corneal staining between baseline and follow-up visits.

A statistically significant reduction in corneal staining grade was observed at visits 2 and 3 (Figure 3). The proportion of eyes with no corneal staining significantly increased at all visits, from 28.3% to 53.4%, 76.8%, and 78.0%, respectively (*p* < 0.001 at all visits vs. baseline). At week 24, 78.4% of eyes with baseline corneal staining showed at least a one-grade reduction.

**Figure 4:**
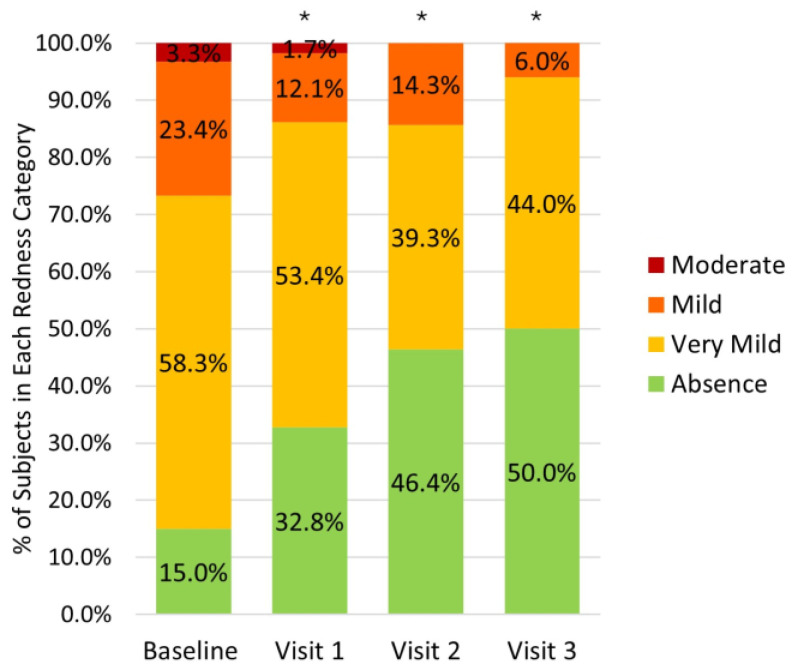
Percentage of eyes in each conjunctival redness category at baseline and follow-up visits. * Indicates *p* < 0.001 for patients with absence of redness between baseline and follow-up visits.

Similarly, a statistically significant reduction in conjunctival redness grade was observed by visit 3 (Figure 4). The proportion of eyes without conjunctival hyperemia increased from 15.0% to 32.8%, 46.8%, and 50.0%, at subsequent visits (*p* < 0.001 at all visits vs. baseline). At week 24, 51.2% of eyes with baseline conjunctival redness showed at least a one-grade reduction.

A statistically significant decrease in IOP was observed from visit 1 onward (1 mmHg), and this change was maintained throughout follow-up (Table 2).

**Figure 5:**
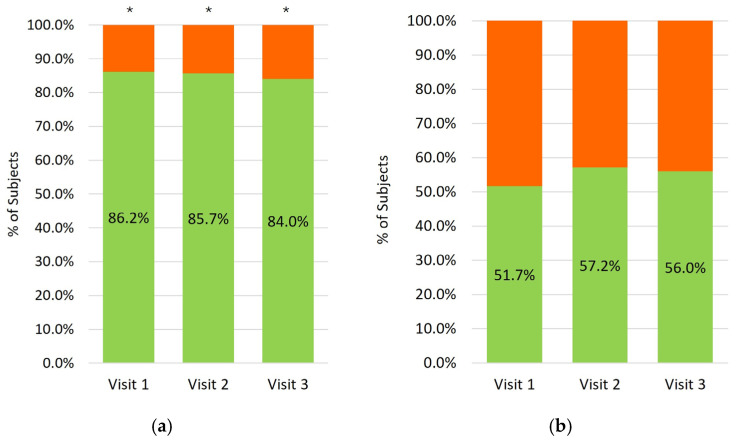
Subjects’ preference regarding comfort of the PF-Dorzolamide 2%-Timolol 0.5% formulation (**a**) and handling of the ophthalmic squeeze dispenser (**b**) at follow-up visits. Green: satisfied or very satisfied, orange: dissatisfied or very dissatisfied. * Indicates *p* < 0.001 between categories.

**Figure 6:**
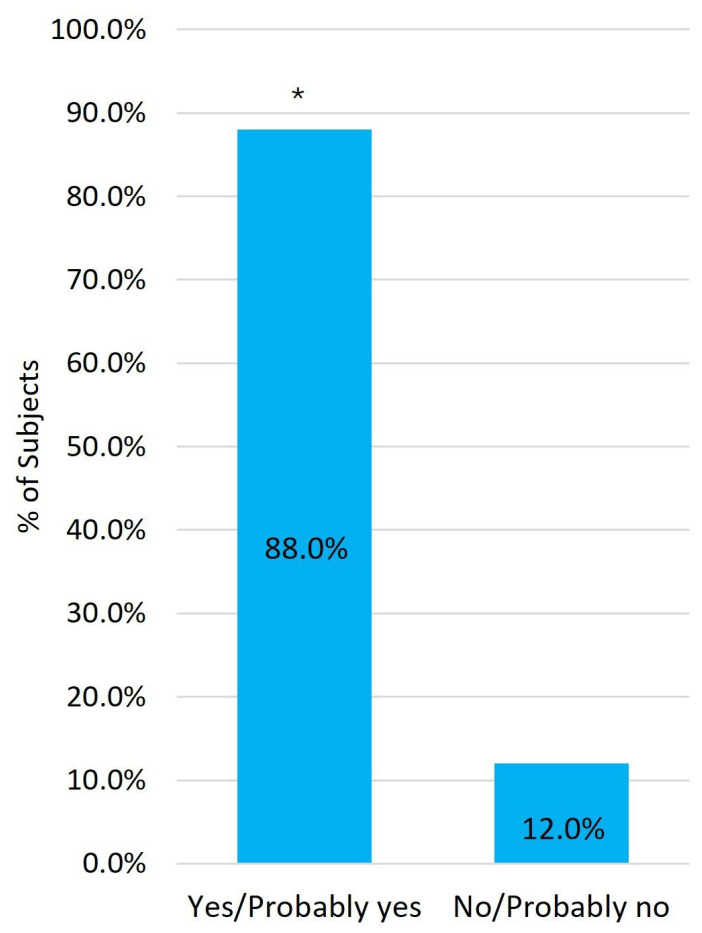
Subjects’ preference for continuing the treatment at week 24. * Indicates *p* < 0.001 between categories.

At all visits, more than 80.0% of patients reported being satisfied or very satisfied with the comfort of PF-DT, and more than 50.0% reported satisfaction with the handling of the ophthalmic squeeze dispenser (Figure 5). At week 24, 88.0% of patients reported willingness to continue PF-DT treatment (Figure 6).

All reported ocular adverse events, including severity, relationship to the study medication, and actions taken, are summarized in Table 3. No treatment-emergent systemic adverse events were reported.

## 4. Discussion

This study allowed for a comprehensive assessment of patients, including objective clinical signs, reported symptoms, and subjective experience. The absence of a washout period reflects routine clinical practice and was intended to capture a real-world treatment transition; however, this design choice also limits the ability to disentangle the specific effects of the treatment change from other potential influences. Previous studies have evaluated switching from preserved to PF glaucoma medications [4,24,25,26,27,28]. However, the present study is the first to demonstrate that glaucoma patients switching from BAK-preserved Dorzolamide 2%-Timolol 0.5% to a PF multidose formulation of Dorzolamide 2%-Timolol 0.5% experience significant improvements in ocular surface disease (OSD) signs and symptoms.

The first large epidemiological study performed in Argentina to evaluate DED revealed that 42.1% of the population suffers from this disease [29]. In our study, the percentage of patients exhibiting DED symptoms decreased to 48.0% after PF treatment, approaching the expected prevalence values in the general Argentine population [29]. The persistence of DED symptoms in some patients after treatment is consistent with the expected prevalence in the general population, indicating that these cases may not be strictly related to glaucoma therapy. This shows a significant improvement in OSD symptoms among glaucoma patients after switching to a PF formulation, supporting the hypothesis that BAK-induced symptoms are at least partially reversible [17,30,31].

Marini et al. also demonstrated that patients receiving glaucoma treatment have twice the risk of developing DED. Furthermore, the prevalence of DED in glaucoma patients was 64.4%, with 16.7% of them presenting severe symptoms [29]. In our study, the percentage of patients with severe symptoms at baseline was also 16.7%, consistent with the study mentioned above [29]. After six months of PF treatment, this percentage was reduced to 4.0%. These results emphasize the potential of PF treatment to significantly reduce OSD symptom severity in glaucoma patients, even when complete resolution is not achieved.

We also demonstrated that removing BAK improved tear film stability, reflected by a significant increase in BUT, and a positive trend in the improvement of Schirmer levels. Since BUT and Schirmer values improved but did not reach normal reference values, we hypothesize that it could take more time to break the vicious inflammatory cycle triggered by BAK. Mucin-producing goblet cells are highly sensitive to BAK toxicity, which may explain why the tear film still shows some degree of instability, even after removing BAK from the treatment [32,33,34]. Sapkota et al. found a positive correlation between goblet cell density and BUT, concluding that goblet cell density affects tear film stability [35]. Unfortunately, the density of goblet cells was not assessed in this study. Likewise, lower Schirmer levels found in glaucoma patients could also be related to meibomian gland dysfunction. Arita et al. proposed that the underlying mechanism may be related to chronic inflammation, which could lead to alterations in the tear film lipid layer composition, increasing tear evaporation rates and ultimately resulting in tear volume reduction [36].

An improvement in the number of patients with normal BUT and Schirmer values was expected with the elimination of BAK toxicity. However, this was not observed, possibly due to the limited duration of the study. Martone et al. found that patients treated with preserved antiglaucoma medications had significantly lower BUT and tear volume compared to those treated with PF therapies. However, theirs was a cross-sectional study where patients had been treated for at least 12 months with the reference medication, whereas ours was a single-arm study of 6 months, where patients on preserved treatment switched to PF therapy [37].

After only 6 months of treatment, the reduction in the percentage of patients with positive staining and hyperemia was 49.1% and 35.0%, respectively, which is consistent with the elimination of cytotoxic stimulus.

Although significant improvements in ocular surface parameters were observed, complete normalization was not achieved during the study period. This may be explained by the chronic nature of ocular surface disease and the long-term prior exposure to preservatives. In addition, it should be acknowledged that the active antiglaucomatous compounds themselves may exert a direct effect on the ocular surface. Together with the relatively limited duration of follow-up, these factors may account for the persistence of residual ocular surface alterations, suggesting that longer treatment periods or adjunctive supportive therapies may be required to achieve full normalization.

It has been previously demonstrated that chronic inflammation related to BAK not only affects the ocular surface but also deeper ocular structures, such as subconjunctival tissues and trabecular meshwork [20,38]. This inflammation may also result from the accumulation of BAK in the trabecular tissues, as shown by experimental pharmacokinetic studies [20]. Previous studies have demonstrated that both switching to PF formulations and intensive ocular surface treatment, such as eyelid hygiene, the use of ocular corticosteroids, PF lubricants, and oral omega-3 supplementation, significantly reduce intraocular pressure levels in glaucoma patients [26,39,40,41].

Although a statistically significant reduction in intraocular pressure of approximately 1 mmHg was observed, this magnitude is small and falls within the known test–retest variability of Goldmann applanation tonometry [42]. It is possible that this is may be caused by improved adherence due to follow-up appointments in a clinical context, better tolerance or greater comfort after switching to PF treatment, among others. Therefore, this finding should be interpreted with caution and cannot be considered clinically meaningful in isolation. However, Leske et al. demonstrated that the risk of glaucoma progression decreases by at least 10% for each millimeter of mercury reduction in IOP [43]. Chamarad et al. identified an association between exposure to preserved antiglaucoma medications and an increased need for glaucoma surgery, suggesting better IOP control with PF treatment [44]. They demonstrated that patients receiving preserved antiglaucoma treatments have an eight times higher risk of requiring surgical intervention compared to those treated with PF medications [44]. This suggests that the sum of small interventions may have a positive impact on the reduction in IOP and ultimately on patients’ quality of life.

In addition to the underlying OSD caused by the topical treatment itself, other factors contributing to poor adherence are the patient’s lack of understanding about the risk of visual loss and their adaptation to handling different available dropper bottles. Modifying these factors could potentially increase patients’ adherence to the treatment [13]. Contrary to our findings, Shedden et al. showed that the removal of BAK from the formulation with a single-dose product does not affect its IOP-lowering efficacy [5]. Considering that our formulation is identical to the one used in Shedden’s study, a hypothesis could be that the observed difference in PIO-lowering effect between BAK-DT and PF-DT may be attributed to patient satisfaction with the multidose dropper. This difference in container type could potentially influence patient compliance, which in turn may impact the hypotensive efficacy of the treatment [45].

Therefore, although the short-term clinical difference between formulations may not be relevant, the use of PF multidose formulations with user-friendly delivery systems could offer a long-term advantage due to improved adherence.

During this study, although only 56.0% of patients expressed satisfaction with the handling of the ophthalmic squeeze dispenser after 6 months of treatment, 88.0% reported a preference for continuing with the PF treatment, highlighting that patients prioritize improvements of ocular symptoms over the challenges of adapting to a new technology.

An important limitation of this study is its open-label, single-arm design, which does not allow discrimination between the true effect of the treatment change and other non-specific influences, such as regression to the mean, increased patient awareness associated with study participation, or the natural course of the disease. In the absence of a control or comparator group, causal inferences cannot be definitively established; therefore, the findings should be interpreted as descriptive of longitudinal changes observed following a treatment switch under real-world clinical conditions. In addition, the inclusion criteria intentionally selected patients with both subjective symptoms and objective signs of ocular surface disease and with well-controlled intraocular pressure at baseline, which introduces a selection bias toward individuals more likely to show improvement after preservative withdrawal. Consequently, the results should not be generalized to the broader glaucoma population without manifest ocular surface disease or to patients with uncontrolled intraocular pressure. In addition, this was an open-label, nonmasked study, with the OSDI as the primary endpoint. As a subjective patient-reported outcome, the OSDI test may be influenced by expectation and reporting bias. However, it was selected because it is a validated and widely accepted measure of ocular surface symptoms, which were central to the study objective, and its interpretation alongside objective clinical signs allowed for a more comprehensive assessment of longitudinal changes. Finally, although improvements in BUT and Schirmer test values were observed, these parameters did not reach normal reference ranges, suggesting that a longer treatment period may be required for full normalization. Despite a dropout rate of 16.7%, the final number of patients completing follow-up met and exceeded the minimum sample size required by the initial power calculation. Therefore, the achieved sample size was sufficient to preserve the planned statistical power for detecting clinically meaningful changes in the primary endpoint. Two patients reported blurred vision, a known and previously reported ocular adverse event associated with the Dorzolamide 2%-Timolol 0.5% fixed combination [46]. With regard to external validity, although the study was conducted in a single country, the clinical context reflects international recommendations that favor PF therapy in patients with glaucoma and ocular surface disease. Therefore, the observed real-world transition scenario is expected to be broadly applicable beyond the local setting.

## 5. Conclusions

In conclusion, considering the known adverse effects of BAK-preserved antiglaucomatous medications on the ocular surface and deeper tissues, the findings of this study suggest that switching to PF formulations may be associated with improvements in ocular surface parameters in patients with ocular surface disease.

## Figures and Tables

**Table 1:** Baseline characteristics.

Characteristic	Value
Patients, n	30
Age, years, mean ± SD	68.8 ± 16.68
Sex, n (%)	
Female	19 (63.3%)
Male	11 (36.7%)
Duration of POAG, months, median (IQR range)	65 (23.3–119.3)
Duration of prior BAK exposure, months, median (IQR range)	65 (23.3–119.3)
Ocular surface disease, n (%)	30 (100%)
Systemic comorbidities, n (%)	
None	11 (36.7%)
Arterial hypertension (HTN)	8 (26.7%)
Hypothyroidism	6 (20.0%)
Dyslipidemia	5 (16.7%)
Hiperchoesterolemia	3 (10.0%)
Diabetes mellitus	2 (6.6%)
Gastritis	1 (3.3%)
Parkinson’s	1 (3.3%)
Hyperuricemia	1 (3.3%)

**Table 2:** Clinical parameters at baseline and follow-up visits: Ocular surface status and intraocular pressure.

	Baseline	Visit 1	Visit 2	Visit 3
OSDI score	21.5 (20.8–28.1)	18.5 (14.1–22.1) ^a^	14.6 (10.4–16.2) ^b^	12.5 (9.8–17.2) ^b^
% Patients with normal OSDI	0 (0–0)	34.5 (12.4–51.7) ^b^	42.9 (23.1–64.1) ^b^	52.0 (31.0–73.1) ^b^
BUT (s)	5.0 (4.7–5.7)	6.0 (5.5–6.7)	7.0 (6.4–7.9) ^b^	7.0 (6.1–7.2) ^a^
Schirmer (mm/5 min)	6.0 (5.9–7.5)	7.0 (7.0–9.3)	8.0 (7.0–9.1)	8.0 (7.0–9.0)
IOP (mmHg)	15.0 (14.5–15.8)	14.0 (13.6–14.8) ^a^	14.0 (13.1–14.6) ^b^	14.0 (12.9–14.4) ^b^

Results are expressed as median (95% CI). Abbreviations: OSDI, Ocular Surface Disease Index; BUT, tear break-up time; s, seconds; min, minutes; IOP, intraocular pressure. ^a^ indicates *p* < 0.05 vs. Baseline (adjusted for Bonferroni correction), ^b^ indicates *p* < 0.01 vs. Baseline (adjusted for Bonferroni correction).

**Table 3:** Safety profile: reported adverse events during treatment.

Adverse Event	Number Un Patients (n)	Severity Grading	Relationship to Study Drug	Action Taken
Hordeolum	1	Mild	Unrelated	No action required; treatment continued
Eye burning	1	Mild	Possibly related	No action required; treatment continued
Blurred vision	2	Mild	Possibly related	Treatment discontinued

## Data Availability

The data supporting this study are not publicly available due to privacy and ethical restrictions related to patient medical records.

## Abbreviations

The following abbreviations are used in this manuscript:

Min	Minutes
S	Seconds
DED	Dry Eye Disease
BAK	Benzalkonium Chloride
OSDI	Ocular Surface Disease Index
BUT	Break-Up Time
GAT	Goldmann Applanation Tonometer
PEEs	Punctate Epithelial Erosions
CCLRU	Cornea and Contact Lens Research Unit
ITT	Intention-to-treat
IOP	Intraocular Pressure
PF	Preservative-free
